# Characterization of a phenotypically severe animal model for human AB-Variant GM2 gangliosidosis

**DOI:** 10.3389/fnmol.2023.1242814

**Published:** 2023-11-30

**Authors:** Natalie M. Deschenes, Camilyn Cheng, Prem Khanal, Brianna M. Quinville, Alex E. Ryckman, Melissa Mitchell, Alexey V. Pshezhetsky, Jagdeep S. Walia

**Affiliations:** ^1^Centre for Neuroscience Studies, Queen’s University, Kingston, ON, Canada; ^2^Department of Pediatrics, Queen’s University, Kingston, ON, Canada; ^3^Centre Hospitalier Universitaire Sainte-Justine Research Centre, Department of Pediatrics, University of Montreal, Montreal, QC, Canada

**Keywords:** NEU3, ABGM2 gangliosidosis, GM2 activator protein, mouse model, central nervous system

## Abstract

AB-Variant GM2 gangliosidosis (ABGM2) is a rare and lethal genetic disorder caused by mutations in the *GM2A* gene that lead to fatal accumulation of GM2 gangliosides (GM2) in neurons of the central nervous system (CNS). *GM2A* encodes a transport protein known as GM2 activator (GM2A) protein, which is essential for degrading GM2 into their GM3 form. ABGM2 presents in infantile-, juvenile-, and adult-onset forms; of the three, the infantile-onset is the most prominent, and by far the most severe, as evidenced by high levels of GM2 accumulation, widespread neurodegeneration, and death by the age of 4. *Gm2a*^−/−^ mice are commonly used as a model of ABGM2. These mice are characterized by phenotypes most representative of predicted adult-onset form of ABGM2, which include moderate GM2 accumulation and mild neurological defects. This mild phenotype has been attributed to compensation by alternative GM2 degradation pathways mediated by sialidase, neuraminidase 3 (NEU3), a pathway that is more prominent in mice than humans. To assess the extent to which NEU3 contributes to GM2 degradation, we generated double knock-out (*Gm2a*^−/−^*Neu3*^−/−^) mice. Compellingly, these mice present with a clinical phenotype resembling that of a more severe ABGM2, including ataxia, reduced mobility and coordination, weight loss, poor body scores, and lethality by 6–7 months. Furthermore, these phenotypes correlate with a dramatic increase in GM2 accumulation in the CNS compared to levels observed in either *Gm2a*^−/−^ or *Neu3*^−/−^ mice. Taken together, these studies, for the first-time, confirm that the mild neurological phenotype of *Gm2a*^−/−^ mice is due to compensatory activity on GM2 catabolism through an alternate breakdown pathway involving NEU3. These studies support the use of double knockout mice as a novel and highly relevant model for pre-clinical drug studies in a more severe form of ABGM2.

## 1 Introduction

Gangliosides are important components of the plasma membrane of vertebrate cells that modulate cell–cell and cell-pathogen signaling by interacting with membrane receptors, adhesion molecules, and ion channels (Sipione et al., 2020; Quinville et al., 2021). These amphipathic molecules are highly enriched in the central nervous system (CNS), and their synthesis and catabolism are tightly regulated. Gangliosides are transported via endocytosis and sorted to lysosomes where they are degraded by hydrolytic enzymes. A negative surface charge and a narrow pH range (3.8–4.6) stimulates the catabolic activity of these enzymes and their activator proteins. Catabolism of gangliosides involves stepwise hydrolysis of their glycan chains by lysosomal exoglycosidases (reviewed by Quinville et al., 2021).

Gangliosides are the main carriers of sialic acid in the CNS and are essential lipids for the maintenance and maturation of the brain and spinal cord. GM1 and GM2 gangliosides (hereafter referred to as GM1 and GM2, respectively) are a type of ganglioside that have a single sialic acid carbohydrate as a head group. Aberrant regulation or function of these gangliosides has been implicated in a number of diseases; these include Parkinson’s disease, Alzheimer’s disease, and a class of disorders known as GM2 gangliosidosis (Quinville et al., 2021).

**Figure 1:**
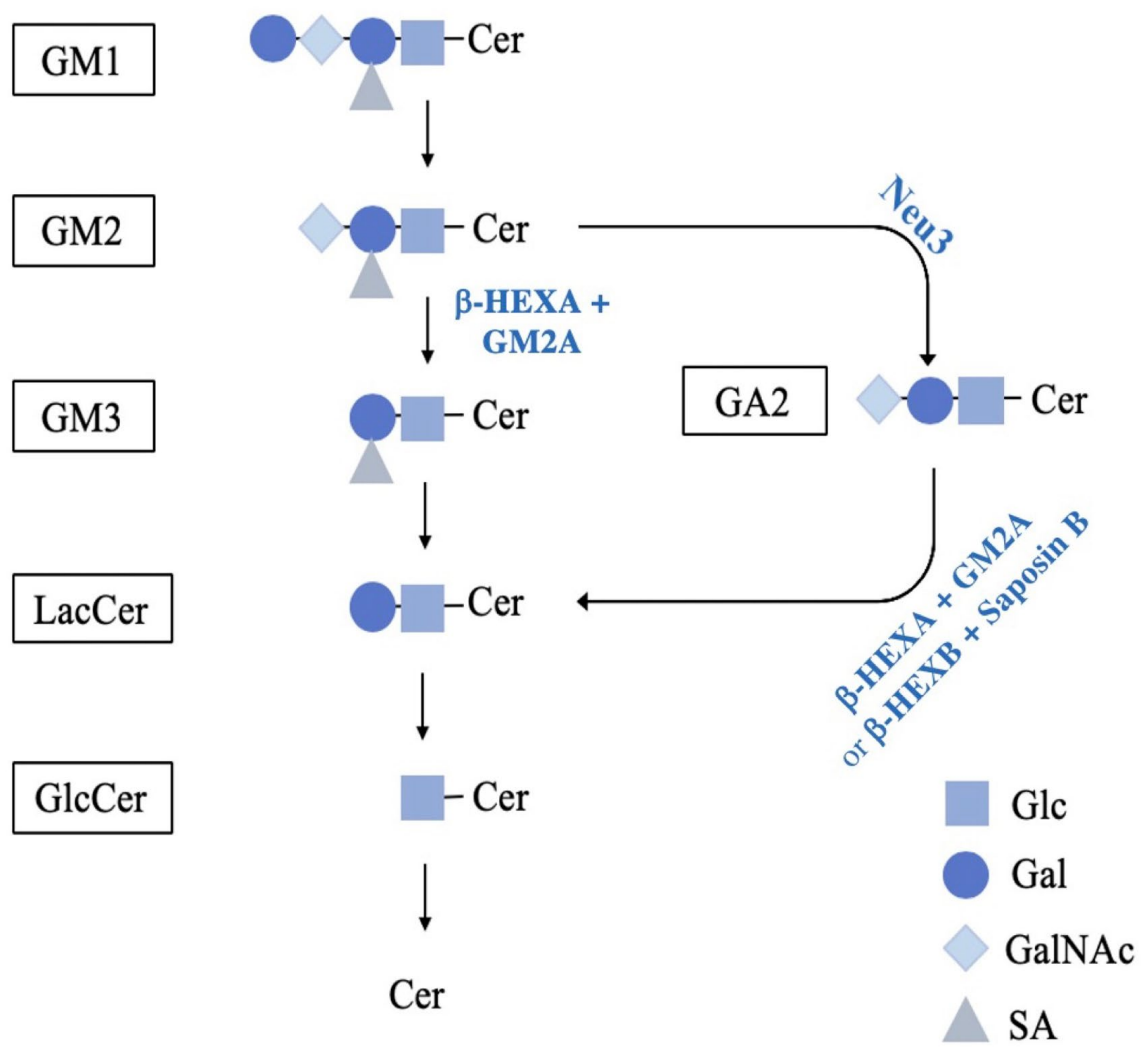
Schematic diagram of the proposed pathway for GM2 catabolism. GM1 breakdown is mediated by GM1-β-galactosidase, cleaving the terminal β-D-galactose with the assistance of GM2A or saposin B, resulting in GM2. GM2 is reduced to GM3 by β-HEXA and GM2A to remove N-acetylgalactosamine. Next, GM3 breakdown is mediated by sialidase and saposin B to remove the sialic acid residue, producing LacCer. Alternatively, GM2 can be hydrolyzed by a murine-specific pathway involving the sialidase NEU3. In this pathway, NEU3 removes the terminal sialic acid residue, resulting in GA2. GA2 is then reduced to LacCer by isoforms β-HEXA and β-HEXB by removing the terminal N-acetylgalactosamine. The galactose is then hydrolysed from LacCer by GM1-B-galactosidase with saposin B or galactosylceramide-β-galactosidase with saposin C into GlcCer. Finally, GlcCer is reduced to ceramide by glucosylceramide-ß-glucosidase with saposin C. (reviewed in Quinville et al., 2021). GM1, GM1; GM2, GM2; GM3, GM3; LacCer, lactosylceramide; GlcCer, glucosylceramide; Cer, ceramide; GA2, glycolipid GA2; Glc, glucose; Gal, galactose; GalNAc, remove N-acetylgalactosamine; SA, sialic acid.

In humans, the hydrolysis of GM2 requires specific synthesis, processing, and combination of β-Hexosaminidase A (β-HEXA), a heterodimer composed of an α and β-subunit, and GM2 activator (GM2A) protein. β-HEXA is a lysosomal enzyme that functions to breakdown GM2 to GM3 by hydrolyzing N-acetylgalactosamine from GM2 (Figure 1). This enzyme requires GM2A, a transport and lipid-binding cofactor that potentiates its activity against GM2. GM2A binds, solubilizes and presents gangliosides for degradation by forming a complex with β-HEXA to mediate the efficient breakdown of GM2 (Conzelmann et al., 1988; Mahuran, 1999). Mutations in the genes encoding the α- (*HEXA*) and β-subunits (*HEXB*), or GM2A (*GM2A*) lead to Tay-Sachs Disease (TSD; OMIM #272800), Sandhoff Disease (SD; OMIM #268800) and AB-Variant GM2 gangliosidosis (ABGM2; OMIM #272750), respectively. Collectively these are known as the GM2 gangliosidosis.

TSD, SD and ABGM2 are a group of severe, autosomal recessive, lysosomal storage disorders characterized by widespread accumulation of GM2 with resultant neuronal apoptosis and early death (Bley et al., 2011). There are three distinct forms of these diseases that are distinguished by their onset, with the most severe being the infantile form, followed by the rarer and milder juvenile- and adult-onset forms. The severity and onset is inversely proportional to the amount of residual β-HEXA or GM2A activity present in affected patients (Conzelmann and Sandhoff, 1991; Leinekugel et al., 1992; Bley et al., 2011). Infantile-onset of these diseases typically manifest between the ages of 3–6 months of age and exhibit the lowest levels of functional β-HEXA or GM2A activity (>2%), resulting in death by 4 years of age (Bley et al., 2011; Sheth et al., 2016). In contrast, the juvenile and adult forms have comparatively higher residual protein activity (5–15%) with time of onset as early as 2 and 21 years of age, respectively. Premature death is evident in juveniles, occurring around 10–15 years of age, while patients with the adult form can live for over 60 years (Neudorfer et al., 2005; Maegawa et al., 2006; Toro et al., 2021). Regardless of the form each patient present with, there is a substantial reduction in quality of life and there is currently no curative treatment available.

The animal model commonly used to study ABGM2 is the *Gm2a*^−/−^ mouse model (Liu et al., 1997). These mice are homozygous for a *Gm2a*^m1R1p^ targeted mutation and thus do not express GM2A protein. As such, they were predicted to phenocopy the severity of pathologies that arise in human infantile ABGM2; however, unlike comparable SD mouse models (Phaneuf et al., 1996), the GM2 gangliosidosis-like pathologies in these mice were unexpectedly less severe. They exhibit moderate levels of GM2 accumulation in the brain (at 20 weeks), mild behavioral and motor dysfunction (e.g., decreased coordination), and a normal life span (Liu et al., 1997). Hence, *Gm2a*^−/−^ mice fail to accurately model the severity of neurological decline and lethality of the infantile- or juvenile-onset forms of human ABGM2. This shortcoming has been postulated to be due to the presence of an alternative, sialidase-mediated GM2 catabolic pathway more prominent in mice than humans (Figure 1). Such a pathway provides an alternative GM2 breakdown route that bypasses or compensates for absent (or weakened) β-HEXA/GM2A-mediated breakdown in *Gm2a*^−/−^ mice (Kolter and Sandhoff, 1998).

Sialidase(s) are capable of hydrolyzing terminal sialic acid from the glycan chains of gangliosides and could potentially convert GM2 to its asialo-GA2 form, which can be further hydrolysed by β-HEXB (an isozyme of β-HEXA) which is present at normal levels in ABGM2 mice (Sango et al., 1995). Three types of sialidases are enriched in mouse neurons: neuraminidase 1 (NEU1), neuraminidase 3 (NEU3) and neuraminidase 4 (NEU4). NEU1 forms a multi-enzyme complex with β-galactosidase and cathepsin A in the lysosome to mediate the breakdown of sialylated glycopeptides and oligosaccharides (reviewed in Monti and Miyagi, 2015). It has strong hydrolyzing activity for GM3 and GD1A, with low activity toward GM2; suggesting that NEU1 is unlikely to mediate an alternate pathway in GM2 catabolism (Timur et al., 2015). NEU3 and NEU4 have high substrate specificity to gangliosides in the presence of an activator protein (Akyıldız Demir, 2019). When *Neu4* was knocked out in the context of *Hexa*^−/−^ mice (which are commonly used as a TSD mouse model), significantly higher GM2 levels, epileptic seizures, and neuronal death was observed (Seyrantepe et al., 2010). However, the extent of pathological abnormalities was not as severe as age-matched SD mouse model (*Hexb*^−/−^) (Sango et al., 1995) and mice were still surviving around 12 months of age (Seyrantepe et al., 2010). This data suggests that *Neu4* is not the only contributor potentiating the pathological severity of GM2 gangliosidosis in mice. Moreover, NEU3 is responsible for about 30% of neuraminidase activity in the brain and for about 50% of activity against gangliosides (Pan et al., 2017). Mice doubly-deficient in *Hexa* and *Neu3* display severe neurological abnormalities, ataxia, tremors and significantly higher GM2 accumulation in the brain (Pan et al., 2017; Seyrantepe et al., 2018). *Neu3*^−/−^ mice also displayed inclusions and vacuoles that are typically observed in lysosomal sphingolipid disorders, and which were not observed in *Hexa^−/-^Neu4^−/−^* mice (Pan et al., 2017). These studies suggested that NEU3 may mediate an alternative degradative pathway that could compensate for disruption of β-HEXA/GM2A-dependent catabolism of GM2. This has led us to hypothesize that ablating *Neu3* in a *Gm2a*^−/−^ mouse background may increase neurological and motor impairment severity and be a more representative model of a severe form of ABGM2. To explore this hypothesis, *Neu3*^−/−^ and *Gm2a*^−/−^ mice were crossbred to generate a *Gm2a*^−/-^*Neu3*^−/−^model. Behavioral and motor phenotypes of *Gm2a*^−/−^*Neu3*^−/−^ mice were then assessed to evaluate their suitability as a more severe animal model to represent human ABGM2. We expected to create a severe phenotype similar to some of the available severe mouse models for GM2 gangliosidosis with HexA deficiency, i.e., SD (*Hexb^−/−^*) (Phaneuf et al., 1996) and double knockout TSD (*Hexa*^−/-^*Neu3*^−/−^) (Seyrantepe et al., 2018) models.

## 2 Materials and methods

### 2.1 Generation of a double knockout mouse

*Gm2a*^−/−^ mice (Liu et al., 1997) were purchased from Jackson Laboratories (Maine, United States). *Neu3*^−/−^ (Yamaguchi et al., 2012) mice obtained from the Centre Hospitalier Universitaire Sainte-Justine Research Centre (Montreal, Quebec, Canada) with a kind permission from Professor T. Miyagi, who generated this strain (Yamaguchi et al., 2012). Mice were bred, housed, and maintained at the Animal Care Facility at Queen’s University (Kingston, Ontario, Canada) as previously described (Liu et al., 1997; Yamaguchi et al., 2012). All experimental procedures were approved by the institutional Animal Care Committee in accordance with the Canadian Council for Animal Care.

*Gm2a*^−/−^*Neu3*^−/−^ colonies were generated by crossing single knockout mouse models (*Neu3*^−/−^ × *Gm2a*^−/−^) to produce F1 generation *Gm2a/Neu3* heterozygotes. Heterozygotes (*Gm2a*^+/−^ *Neu3*^+/−^) were crossed to produce F2 double knockout progeny (*Gm2a*^−/-^*Neu3*^−/−^) in proportions consistent with Mendel’s Law of Independent Assortment.

### 2.2 Genotyping

Genotyping was performed on DNA extracted from ear notches collected from the mice at or before 21 days of age. DNA digestion was carried out using Q5^®^ High-Fidelity 2X Master Mix (New England BioLabs Ltd., Ontario, Canada). Samples were then prepared for polymerase chain reactions (PCR). The following primers were used to detect *Gm2a* and *Neu3*:

*Gm2a* Mutation Forward 5′-CTTGGGTGGAGAGGCTATTC-3′;
*Gm2a* Mutation Reverse 5′-AGGTGAGATGACAGGAGATC-3′;
*Gm2a* WT Forward 5′-TACCTACTCACTACCCACGAGC-3′;
*Gm2a* WT Reverse 5′-ACACAGAAGAAGAGGCCTGC-3′.
*Neu3* Forward 5′- GCTCTACCCCATTCTACATCTCCAGAC-3′.
*Neu3* Reverse: 5′- GTGAGTTCAAGAGCCATGTTGCTGATGGTG-3′.
*Neu3* Neo Cassette Forward: 5′- TCGTGCTTTACGGTATCGCCGCTCCCGATT-3′.

### 2.3 Behavioral testing

A series of behavioral tests were conducted to characterize potential physical impairments in double knockout mice. Testing began at 8 weeks of age and continued every 4 weeks, until 16 weeks of age. After this period, testing was conducted bi-weekly until endpoint. Frequency of behavioral testing was increased to weekly, starting at 16 weeks of age, to determine the rate of behavioral decline. Two behavioral parameters were tested. These included an Open Field Test (OFT, ActiMot, TSE systems, Berlin, Germany) to evaluate gross locomotor activity (Osmon et al., 2018), and a RotaRod test (RR; IITC Life Sciences, California, United States) to evaluate motor coordination and balance (Osmon et al., 2018).

For the OFT, mice were placed in a bordered arena (40 cm × 40 cm) for 5 min and were allowed to roam freely. Resting time, distance traveled, and speed during this time was recorded (Osmon et al., 2018).

For the RR, mice were placed on elevated rotating cylinders that accelerated from 4 to 40 rotations per minute (rpm) over 5 min. When the mouse fell, it off balanced a beam below them, which signaled the end of the test. Each mouse was tested on the RR apparatus three times per time point, with a minimum of 10 min of rest between each trial. Latency to fall, end rotations per minute (rpm) and distance traveled were recorded (Osmon et al., 2018).

### 2.4 Euthanization

*Gm2a^−/-^Neu3^−/−^* mice were euthanized at their humane endpoint and all other cohorts were euthanized between 27 and 30 weeks of age. A humane endpoint is defined as the point at which a mouse loses 15% of its peak weight, or when a mouse is unable to right itself (which indicates a reduced quality of life). Mice are sacrificed by CO_2_ asphyxiation according to the Animal Care Committee guidelines. Organs were perfused with phosphate-buffered saline (PBS) and collected thereafter, for future biochemical and histological analysis.

Cardiac puncture on euthanized mice was used to collect blood to assess serum protein levels. Organ perfusion with PBS was carried out following cardiac puncture. Gross organs and CNS tissue were collected for Western blot analysis, ganglioside accumulation assays and histological imaging. Regions of the brain and spinal cord dissected for tissue collection and sectioning are shown in Supplementary Figure 1.

### 2.5 Western blot

Protein extracts from murine livers were separated by SDS-PAGE and transferred to a nitrocellulose membrane (Bio-Rad Laboratories, California, United States). Non-specific binding sites were blocked with a 5% skim milk tris-buffered saline (TBS) solution for 1 h. Membranes were then incubated with primary antibodies against GM2A (Taysha Gene Therapies, Texas, United States; TSHA081821; 1:1000), α-subunit of β-HEXA (Abcam, Cambridge, United Kingdom; ab189865; 1:1000), β-subunits of β-HEXA (Thermo Fisher Scientific, Massachusetts, United States; 16229-1-AP; 1:1000) or β-actin (Sigma-Aldrich, Missouri, United States; A5316; 1:5000) overnight in 5% skim milk. Membranes were then washed three times in TBS-Tween-20 (TBST) for 10 min prior to incubation with secondary antibody for 1 h at room temperature. Secondary antibodies included rabbit anti-Goat-HRP (Sigma-Aldrich, Missouri, United States; AP106P; 1:5000) for GM2A, and goat anti-mouse-HRP (Sigma-Aldrich, Missouri, United States; A5278; 1:5000) for β-actin. Membranes were washed again in TBST and then incubated with a horse-radish-peroxidase solution (Millipore Sigma, Massachusetts, United States). Membranes were visualized by chemiluminescence (Bio-Rad Laboratories, California, United States). GM2A and β-actin were quantified through densitometry using IMAGE J Software (National Institute of Health, Maryland, United States).

### 2.6 Ganglioside storage assay

A modified Folch extraction was used as described in Osmon et al. (2018). Briefly, mid-sections of the brain were taken to be representative of whole murine brain. Each mid-section was sonicated and transferred to a glass tube (UltiDent Scientific Inc., Quebec, Canada). Next, a series of methanol and chloroform dilutions were added to the sample and then flowed through a column (Phenomenex, California, United States) to collect gangliosides. Samples were eluted and added to a thin layer chromatography plate (TLC; MilliporeSigma, Massachusetts, United States) for development. The plate was placed in a tank containing chloroform, methanol, and calcium chloride. The plate was developed in the tank for several hours to allow individual gangliosides to separate, and then sprayed with a drying solution made up of orcinol and sulfuric acid. Individual gangliosides were quantified by densitometry by comparing the intensity of the GM2 bands against an internal control (GD1A) using IMAGE J software (National Institute of Health, Maryland, United States). A TLC Monosialoganglioside mixture (MJS Biolynx Inc., Ontario, Canada) and *Gm2a*^−/−^ mid-section samples were used as controls. GD1A was used as an internal control as it is a constant presence in mice, throughout adulthood (Yu et al., 2012).

### 2.7 Hexosaminidase activity assay

β-HEXA activity assays were performed as previously described (Tropak et al., 2004, 2007). Briefly, serum was collected via cardiac puncture and then diluted 1:35 in citrate phosphate buffer (CP Buffer). A portion of the serum was heat inactivated (HI) at 52°C to destabilize β-HEXA, leaving only β-HEXB active in these samples. Next, the substrate 4-methylumbelliferyl-2-acetamido-2-deoxy-β-D-glucopyranoside (MUG; Toronto Research Chemicals, Ontario, Canada) was added to both normal and HI serum samples. Β-galactosidase activity was used as an internal control, which was measured using 4-methylumbelliferyl-β-D-galactopyranoside (MUGal) (MilliporeSigma, Massachusetts, United States).

MUG and MUGal activity were assessed by incubating serum samples at 37°C in a 3.2 mM MUG or MUGal substrate solution. After 1 h, enzyme activity was stopped with 0.1 M 2-amino-2-methyl-1-propanol (AMP). MUG and MUGal activity were estimated based on a 4-methylumbelliferone (4-MU) standard curve, which ranged from 0.73 nM to 130 μM. Activity was detected by colorimetric intensity at an excitation wavelength of 365 nm and an emission wavelength of 450 nm on a Varioskan^™^ microplate reader (Thermo Fisher Scientific, Massachusetts, United States). HexA activity was calculated by subtracting HI sample activity (representative of HexB activity) from total MUG activity (all hexosaminidases), after being normalized to MUGal concentrations.

### 2.8 Histology

Portions of the mid- and caudal-sections of the brain were fixed in 4% paraformaldehyde (PFA) for 24–48 h and then transferred to 70% ethanol for another 24–48 h. Samples were then transferred to PBS and shipped to The Center for Phenogenomics (Toronto, Ontario, Canada) for slide preparation and staining with hematoxylin and eosin, or with an anti-GM2 antibody (a gift from Kyowa Hakko Kirin Co. Ltd., Tokyo, Japan). Ganglioside accumulation was quantified using QuPath Software (Belfast, Ireland).

### 2.9 Statistical analysis

All statistical analyses were performed with GraphPad PRISM 9.3.1. Statistical testing for all behavioral modalities were performed using two-way repeated measures analysis of variance (ANOVA) with Tukey *post hoc* test to compare cohorts across all timepoints. Ganglioside accumulation and hexosaminidase activity were analyzed by a one-way ANOVA with a Tukey *post hoc* test for multiple pairwise comparisons. A Kaplan Meier curve was used to present the survival patterns of each cohort, which was analyzed with a Log-Rank (Mantel-Cox) test to assess significant survival differences. A nonlinear regression (curve fit) was used to determine if there was a correlation between symptom onset and age of death.

## 3 Results

### 3.1 Protein and enzyme activity altered in *Gm2a^−/−^Neu3^−/−^* mice

**Figure 2:**
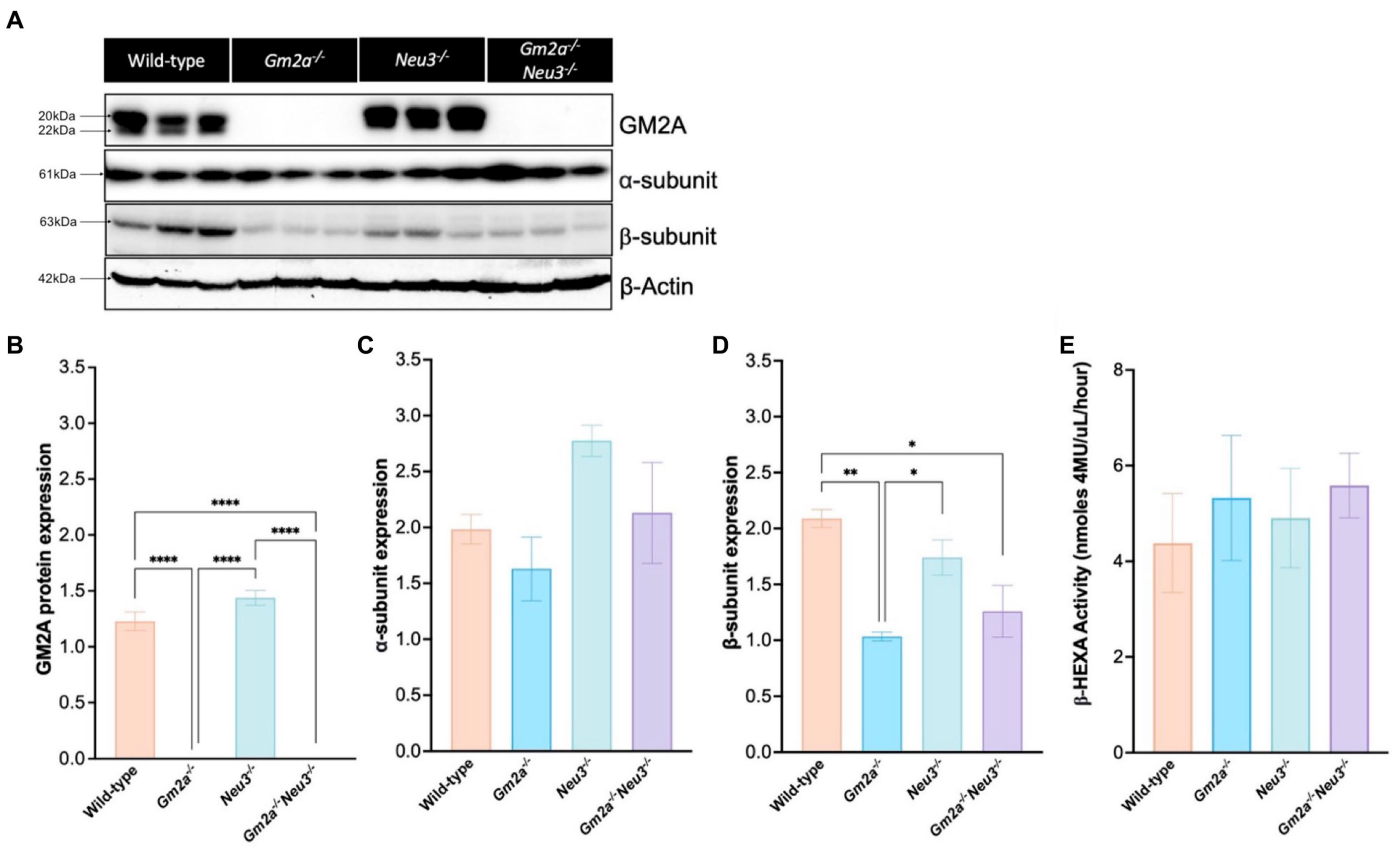
Protein expression and enzyme activity in *Gm2a*^−/−^*Neu3*^−/−^ mice. **(A)** Representative Western blot analysis of GM2A protein and the α-and β-subunits of β-HEXA expression in murine brains. Mid-sections of the brain were dissected from *Gm2a*^−/−^*Neu3*^−/−^ mice at humane endpoint and compared to brain sections extracted from controls (*Gm2a*^−/−^*Neu3*^−/−^ and wild-type) that were euthanized between 27 and 30 weeks of age. Bands migrating at ~20 kDa or 22 kDa depict the mature and precursor forms of GM2A, respectively. Bands migrating at 61 kDa depict the α-subunit of β-HEXA and those migrating at 63 kDa depict the β-subunit of β-HEXA. β-actin was utilized as an internal control and migrates at 42 kDa. **(B)** Quantification of GM2A protein signal in the Western blot. The intensity of the mature and precursor forms of GM2A were taken as a representation of total GM2A protein signal and were normalized to β-actin intensity. Intensities were quantified by densitometry analysis. *Gm2a*^−/−^*Neu3*^−/−^ and *Gm2a*^−/−^ mice had significantly lower (negligible) GM2A protein expression compared to *Neu3*^−/−^ and wild-type mice [*F*_(3,8)_ = 211.7, *p* < 0.0001 (****)]. **(C)** Quantification of α-subunit expression in the Western blot, normalized to β-actin intensity. No significant differences in subunit expression were observed in any of the cohorts [*F*_(3,8)_ = 2.85, *p* > 0.05]. **(D)** Quantification of B-subunit expression in the Western blot, normalized to β-actin intensity. β-subunit expression was significantly lower in *Gm2a*^−/−^*Neu3*^−/−^ and *Gm2a*^−/−^ compared to wild-type mice [*F*_(3,8)_ = 10.40, *p* < 0.0170 (**) and *p* < 0.0043 (*), respectively]. *Gm2a*^−/−^ mice also had significantly less β-subunit expression compared to *Neu3*^−/−^ mice [*F*_(3,8)_ = 10.40, *p* < 0.0382 (*)]. **(E)** Total β-HEXA was quantified from a Hexosaminidase assay using mouse serum at respective endpoints. β-HEXA was normalized to β-galactosidase, an internal control. There were no significant differences noted in enzyme activity between any of the cohorts [*F*_(3,17)_ = 0.24, *p* > 0.05]. Data are expressed as mean ± SEM. All experiments included *n* = 3 per cohort.

A Western blot was performed to confirm the absence of GM2A protein and to determine the effect of knocking out *Neu3* on the expression of the α- and β-subunits in the brain (Figure 2A). As expected, GM2A protein was absent in *Gm2a*^−/−^ and *Gm2a*^−/-^*Neu3*^−/−^ mice and unaffected in wild-type and *Neu3*^−/−^ mice (Figures 2A,B). Expression of the α-subunit was not impacted by knocking out *Neu3* in *Gm2a^−/−^* mice, and there were relatively equal levels of the α-subunit between all cohorts (Figure 2C). Expression of the β-subunit in *Gm2a*^−/-^*Neu3*^−/−^ mice was significantly lower than wild-type mouse brains (Figure 2D). A similar trend was seen in *Gm2a*^−/−^ mice, which had reduced expression of the β-subunit compared to wild-type and *Neu3^−/−^* mice (Figure 2D). To determine the correlation between expression of each subunit and β-HEXA activity, a hexosaminidase activity assay was conducted on mouse serum (Figure 2E). Here, we saw no significant differences in β-HEXA activity in any of the cohorts, regardless of the slightly altered expression levels in the subunits observed in some of the cohorts.

### 3.2 The life span of *Gm2a^−/−^Neu3^−/−^* mice is significantly reduced

**Figure 3:**
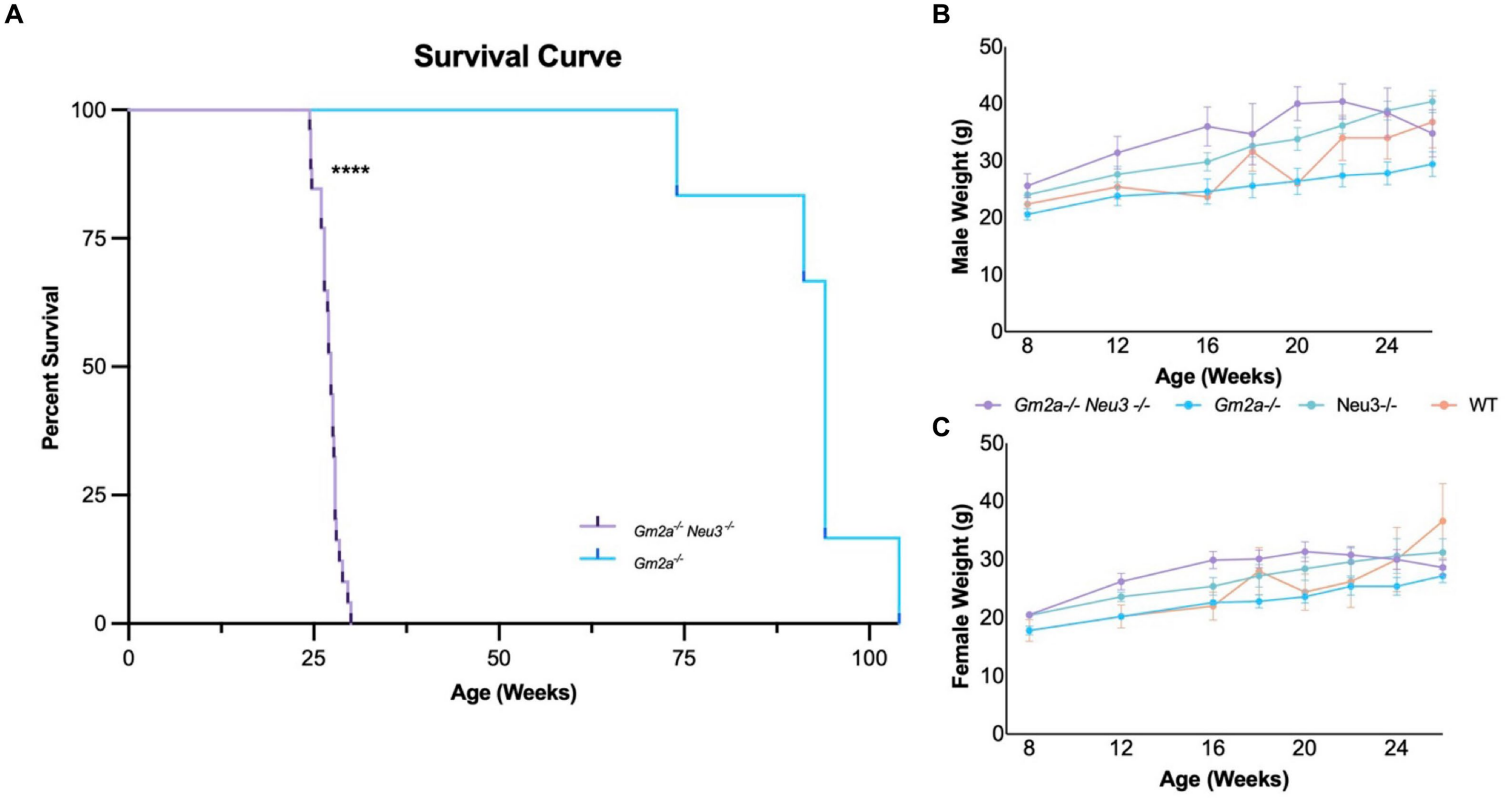
Survival and weight of *Gm2a*^−/−^*Neu3*^−/−^ mice. **(A)** Kaplan–Meier survival curve indicates that the survival of *Gm2a*^−/−^*Neu3*^−/−^ mice is significantly reduced compared to *Gm2a*^−/−^ mice of *Gm2a*^−/−^*Neu3*^−/−^ [*p* < 0.0001 (****); *n* = 29 *Gm2a*^−/−^*Neu3*^−/−^; *n* = 6 *Gm2a*^−/−^; Log-Rank (Mantel Cox) test]. *Gm2a*^−/−^*Neu3*^−/−^, 27.0 ± 1.5 weeks; *Gm2a*^−/−^*Neu3*^−/−^; 91.9 ± 9.8; Age: [avg ± SD]. To evaluate weight differences of **(B)** males [*F*_(3,21)_ = 2.717; *p* > 0.05], and **(C)** females [*F*_(3,21)_ = 1.654; *p* > 0.05], mice were weighed monthly from 8 weeks until their respective endpoints (4 cohorts; *n* = 10/sex, *Gm2a*^−/−^*Neu3*^−/−^; *n* = 5/sex *Gm2a*^−/−^*Neu3*^−/−^, and wild-type). Body weight progressively increases with age, in all cohorts, until 20 weeks of age. At 20 weeks of age *Gm2a*^−/−^*Neu3*^−/−^ mouse weights begin to decline, while others continue to increase. Data are expressed as mean ± SEM.

*Gm2a^−/−^Neu3^−/−^* mice were generated by interbreeding singly knocked out *Neu3* (Yamaguchi et al., 2012) and *Gm2a* (Liu et al., 1997) mice. Kncokout of *Gm2a* and *Neu3* was confirmed by PCR (Supplementary Figures 2A,B). As discussed above, *Gm2a^−/−^* mice exhibit mild neurological and behavioral phenotypes, including normal lifespans (Liu et al., 1997); whereas *Neu3^−/−^* mice are relatively asymptomatic with respect to neurological and behavioral phenotypes (Seyrantepe et al., 2010, 2018). Consistent with previous reports (Liu et al., 1997), we confirm that *Gm2a^−/−^* mice have a normal life span (Deschenes et al., 2023). However, *Gm2a^−/−^Neu3^−/−^* mice had significantly shortened lifespans compared to *Gm2a^−/−^* counterparts (Figure 3A). Double knockout mice lived an average of 27.0 ± 1.5 (mean ± SD) weeks, compared to *Gm2a^−/−^* mice, which lived a relatively normal lifespan that averaged 91.9 ± 9.8 weeks. These results demonstrate that *Neu3* gene in *Gm2a* gene deficient backgrounds reduce lifespans to an extent that may be representative of a more severe form of GM2 gangliosidosis, than the previously utilized animal model (*Gm2a^−/−^* mice).

The newly developed mouse strain exhibited a significantly reduced average lifespan of 27 ± 1.5 weeks (range from 24.43 to 30.0 weeks), compared to *Gm2a^−/−^* mice (91.9 ± 9.8 weeks) (Figure 3A). *Gm2a^−/-^Neu3^−/−^* mice exhibited a range of physical impairments indicative of disease manifestation (Supplementary Figure 3B). A positive correlation between the onset of symptoms of disease manifestation and age of death was observed (Supplementary Figure 3A). Depending on symptom severity, *Gm2a^−/−^Neu3^−/−^* mice lived, on average, for 1.5–3 weeks after the appearance of the first signs of the disease. Moreover, the earlier the symptoms appeared, the earlier the humane endpoint was reached (see *Methods: Euthanizations*). The predominant symptoms indicative of disease included tremulous movement and ataxic gait (Supplementary Figure 3B); these began as early as 21 weeks of age in some mice, while others were relatively asymptomatic until 28 weeks of age. In addition, *Gm2a^−/−^Neu3^−/−^* mice began to lose weight around 20–22 weeks of age with at least 66.7% of them losing over 15% of their peak body weight at humane endpoint (Figures 3B,C). Other symptoms included seizures (4.2%), eye infections (16.7%), and death (8.3%) although these were of substantially less predominance (Supplementary Figure 3B). Four *Gm2a^−/−^Neu3^−/−^* mice experienced hindlimb paralysis around 9–12 weeks of age; however, they have not been included in any of the analyses as we were unable to confirm the cause. Although, this is a classic symptom observed in the SD mouse model (Phaneuf et al., 1996).

### 3.3 Coordination and balance are severely impaired in *Gm2a^−/−^Neu3^−/−^* mice

**Figure 4:**
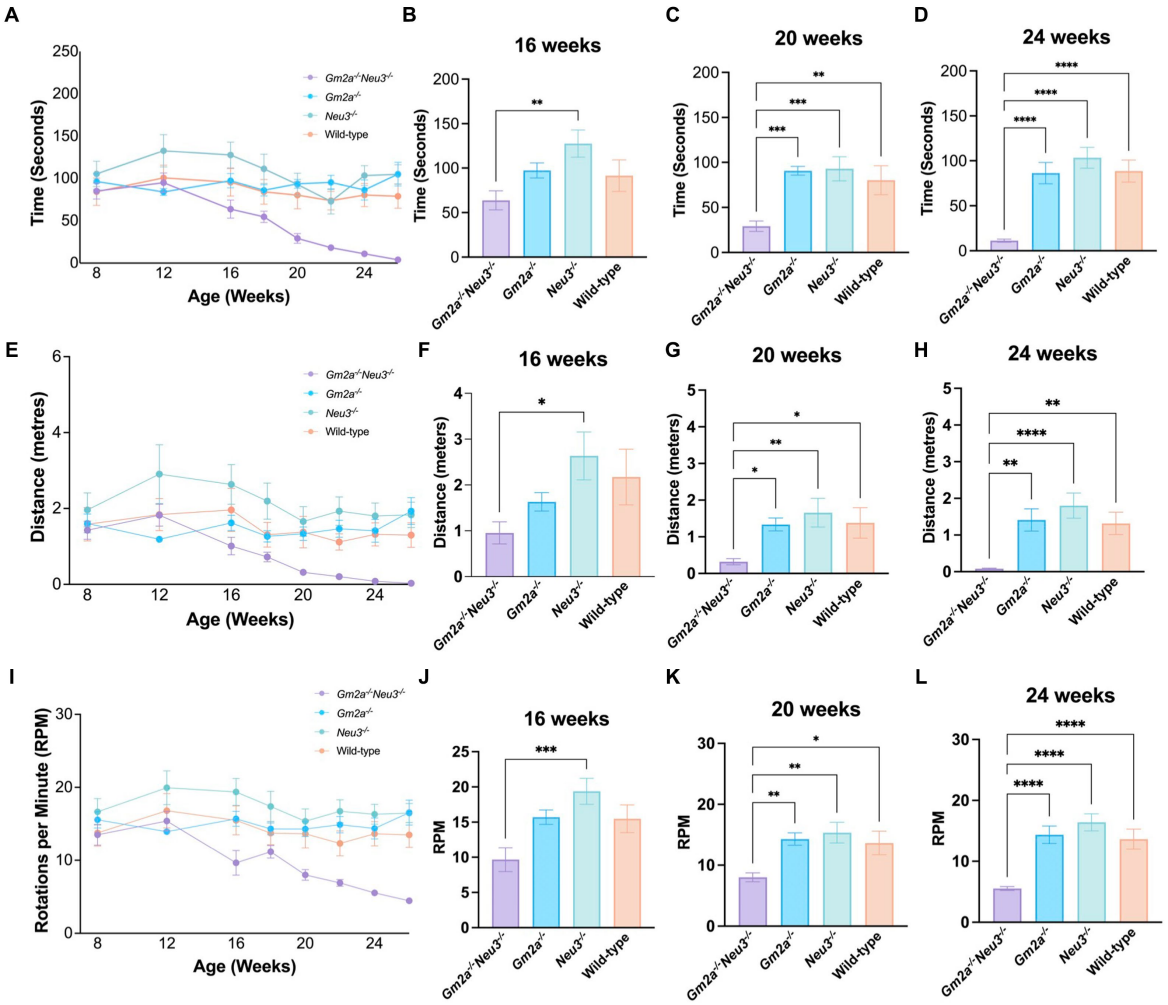
Behavioral test for coordination. Motor function as assessed by RR tests is severely compromised in *Gm2a*^−/−^*Neu3*^−/−^ (*n* = 20) mice compared to age-matched single knockout and wild-type cohorts (*n* = 10 per cohort). To evaluate coordination and balance, RR testing was conducted between 8 and 26 weeks of age. Three parameters were assessed: **(A–D)** latency to fall, **(E–H)** distance traveled, and **(I–L)** end RPM. Data are expressed as mean ± SEM of the indicated parameter. The *Gm2a*^−/−^*Neu3*^−/−^ cohort performed significantly worse, compared to the other cohorts, in all parameters, beginning at 16 weeks of age and progressively declining until 26 weeks of age [latency to fall: *F*_(3,41)_ = 14.81, *p* < 0.0001; distance traveled: *F*_(3,41)_ = 7.091, *p* < 0.0006; end RPM: *F*_(3,41)_ = 12.45, *p* < 0.0001]. *p < 0.05; **p < 0.01; ***p < 0.001; ****p < 0.0001.

Coordination and balance were assessed on a RR apparatus which measures the time that elapses before a mouse falls off an elevated rotating cylinder. Three RR parameters were assessed, including latency to fall, distance traveled, and end RPM. All cohorts tested on this apparatus performed comparably at approximately 8 weeks of age but started to deviate at around 12 weeks of age (Figures 4A,E,I). After 12 weeks of age, *Gm2a^−/−^Neu3^−/−^* mice exhibited a downward trend in performance for all three RR parameters assessed. These trends became significant for all 3 parameters by 20–24 weeks of age and remained significant at later time points (Figures 4B–D,F). By 24 weeks of age, most of the *Gm2a^−/−^Neu3^−/−^* mice were incapable of staying on the rotating cylinder at all. This trend was consistent across all other RR parameters. This progressive decrease in performance on RR with the *Gm2a^−/-^Neu3^−/−^* mice was not observed in other cohorts, suggesting that knocking out *Neu3* in *Gm2a^−/−^* mice imparts coordination and balance defects that are absent in *Gm2a^−/−^*. In addition, *Gm2a^−/−^* mice appear to perform similarly to the wild-type cohort, which opposes a previous paper that characterizes this model (Liu et al., 1997).

### 3.4 Locomotion is severely impaired in *Gm2a^−/−^Neu3^−/−^* mice

**Figure 5:**
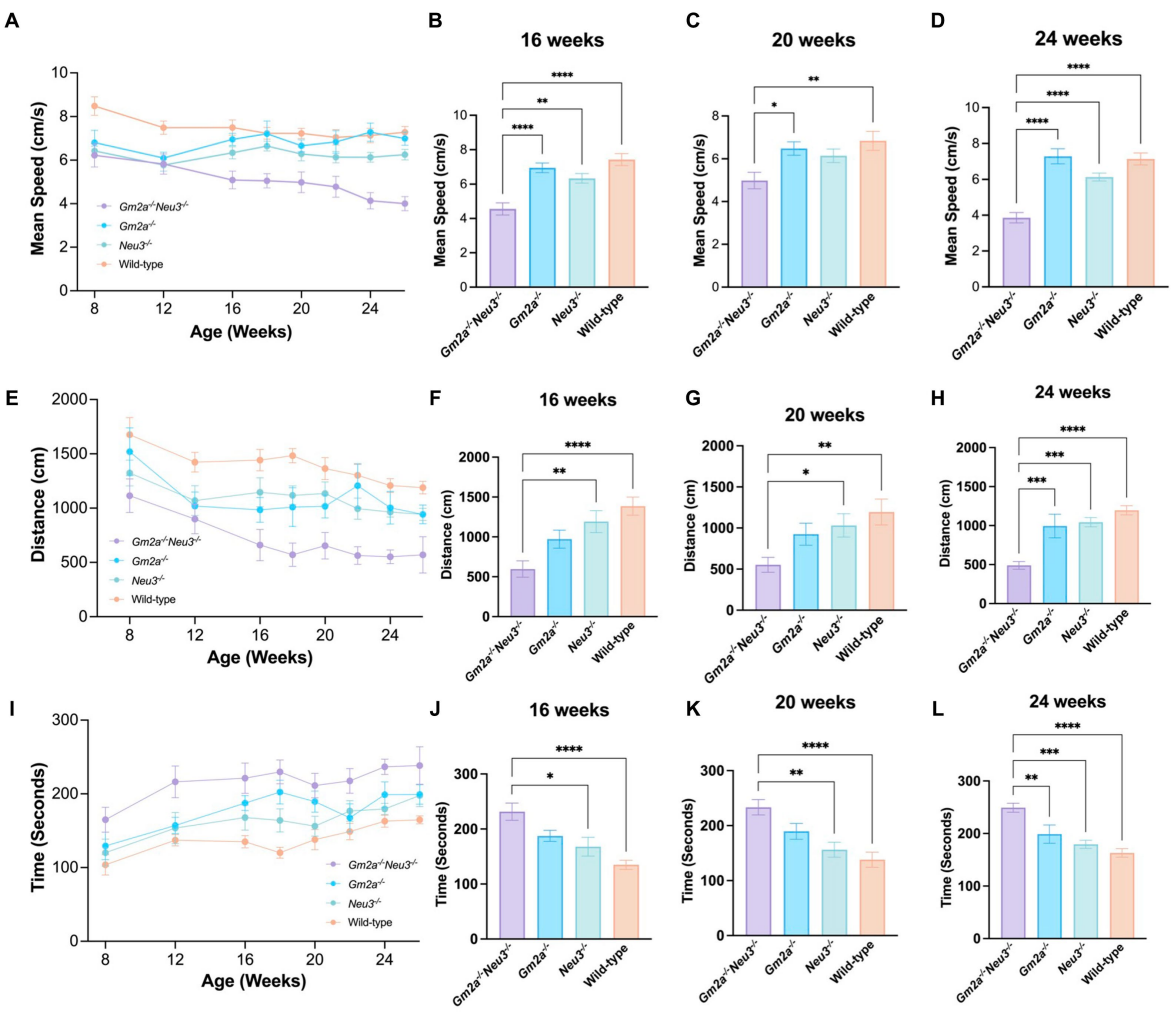
Behavioral test for general locomotion. Motor function as assessed by OFT tests is severely compromised in *Gm2a*^−/−^*Neu3*^−/−^ mice (*n* = 20) compared to age-matched single knockout and wild-type cohorts (*n* = 10 per cohort). To evaluate coordination and balance, OFT testing was conducted between 8 and 26 weeks of age. Three parameters were assessed: **(A–D)** mean speed, **(E–H)** distance traveled, and **(I–L)** resting time. Data are expressed as mean ± SEM of the indicated parameter. The *Gm2a*^−/−^*Neu3*^−/−^ cohort exhibited significantly worse general locomotion, assessed by the OFT, compared to the other cohorts, in all parameters, beginning at 16 weeks of age and progressively declined until 26 weeks of age [mean speed: *F*_(3,41)_ = 18.61, *p* < 0.0001; distance traveled: *F*_(3,36)_ = 14.81, *p* < 0.0001; resting time: *F*(3,36) = 11.31, *p* < 0.0001]. *p < 0.05; **p < 0.01; ***p < 0.001; ****p < 0.0001.

Disparities in motor function were much more apparent between cohorts when evaluated by the OFT, with differences in the *Gm2a^−/−^Neu3^−/−^* mice beginning as early as 8 weeks of age (Figures 5A,E,I). For the OFT parameters evaluated—mean speed, distance traveled, and resting time—the performance of *Gm2a^−/−^* and *Neu3^−/−^* mice were similar, indicating that neither single gene deficiency by itself significantly impacts motor function. Remarkably however, *Gm2a^−/−^Neu3^−/−^* mice exhibited significantly reduced scores for the mean speed and distance traveled parameters of the OFT when compared to all the cohorts (Figures 5B–D,F). Correspondingly, *Gm2a^−/−^Neu3^−/−^* mice resting time was significantly longer than the other cohorts (5B-5D). These differences became significant at 8–12 weeks of age and persisted through all time points. Furthermore, motor impairments in *Gm2a^−/−^Neu3^−/−^* mice grew progressively worse as indicated by their OFT motor scores, which increasingly diverged from the scores of other cohorts over time (Figures 5A,E,I). These data clearly demonstrate that double knockout mice have impaired gross locomotor activity beginning very early in life.

### 3.5 GM2 accumulates in *Gm2a^−/−^Neu3^−/−^* mice to levels representative of a more severe form of ABGM2

GM2 is a breakdown product of GM1 in the ganglioside catabolic pathway, which then continues to be metabolized in a stepwise fashion into glucosylceramide (GlcCer). This process involves the intermediary products GM3 and LacCer (Figure 1). Importantly, a branching pathway from GM2 to GA2 and then LacCer has also been identified as an alternative catabolic route for GM2 breakdown (Sango et al., 1995; Figure 1). Hence, GlcCer is a key convergence point for two GM2 breakdown pathways and its levels are an important marker of total GM2 breakdown activity.

**Figure 6:**
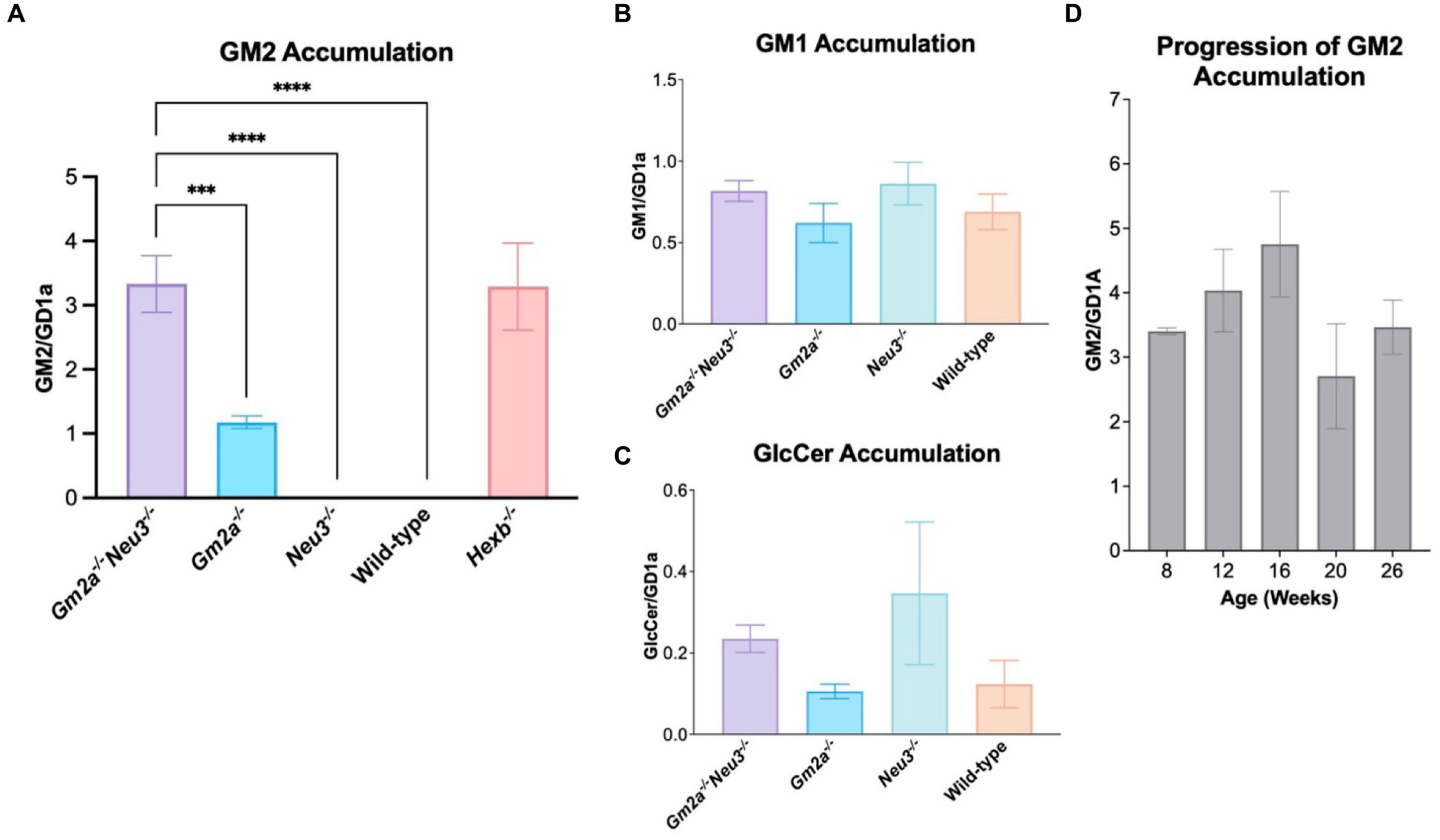
Ganglioside storage assay. **(A)** GM2 accumulation in the mid-section of mice brains at endpoint (24–30 weeks of age), expressed as a function of GDla, an internal control. Overall, *Gm2a*^−/−^*Neu3*^−/−^ mice (*n* = 13) store significantly more GM2 than *Gm2a*^−/−^ (*n* = 9), *Neu3*^−/−^ (*n* = 10), and wild-type mice (*n* = 10) [*F*_(4,51)_ = 28.78, *p* < 0.002 (***), *p* < 0.00001 (****), and *p* < 0.00001 (****), respectively]. Accumulation was comparable between *Gm2a*^−/−^*Neu3*^−/−^ (*n* = 13) and *Hexb*^−/−^ age-matched mice (*n* = 8). **(B)** GM1 accumulation in the mid-section of mice brains, also expressed as a function of GD1a. No significant differences were seen in any of the cohorts [*n* = 3 per cohort; *F*_(3,16)_ = 1.045, *p* > 0.05]. **(C)** GleCer accumulation in the mid-section of mice brains, expressed as a function of GD1a. No significant differences were seen in any of the cohorts [*n* = 3 per cohort; *F*_(3,16)_ = 1.407, *p* > 0.05]. Although, *Gm2a*^−/−^*Neu3*^−/−^ and *Neu3*^−/−^ have an observable increase in GlcCer compared to *Gm2a*^−/−^ and wild-type mice. **(D)** Progression of GM2 accumulation was examined in mice from 8 to 26 weeks of age in the mid-section of *Gm2a*^−/−^*Neu3*^−/−^ brains [*n* = 3 per cohort; *F*_(4,10)_ = 1.530, *p* > 0.05]. Data are expressed as mean ± SEM.

GM1, GM2, GM3 and GlcCer were extracted and quantified using TLC. Quantitative analysis of glycolipids was carried out on the mid-section of brains, which are taken as representative of the whole murine brain. The brains were collected at humane endpoints for *Gm2a^−/−^Neu3^−/−^* mice and at 26–30 weeks of age for all other cohorts. Interestingly, we were unable to detect GM2 accumulation in *Neu3^−/−^* brain tissue indicating that GM2 catabolic efficiency is unimpacted by knocking out the NEU3-mediated route of GM2 breakdown (Figures 1, 6A), likely because GM2 is more efficiently catabolized by β-HEXA/GM2A-mediated pathway. These results were consistent with a previous study (Seyrantepe et al., 2018). In contrast, appreciable GM2 accumulation was detected in the brains of *Gm2a^−/−^* mice, suggesting that GM2 breakdown is impaired, and confirms that GM2A co-factor activity for the β-HEXA enzyme is involved in GM2 degradation. However, knocking out *Neu3* in *Gm2a^−/−^* backgrounds resulted in a striking increase (approximately 3- to 4-fold) in GM2 levels relative to *Gm2a^−/−^* mice. This demonstrates that significantly less GM2 is degraded in *Gm2a^−/-^Neu3^−/−^* mice than in *Gm2a^−/−^* mice (Figure 6A). This also indicates that the NEU3-mediated pathway may be compensating for impaired GM2 breakdown in *Gm2a^−/−^* mice, and that in the absence of this compensation, GM2 accumulation is significantly higher. The levels of other gangliosides quantified remain unchanged in all cohorts (Figures 6B,C). Importantly, the levels of GM2 accumulation in the double knockouts parallel the severity of GM2 accumulation observed in a *bona fide* model of infantile SD cohort of age-matched *Hexb^−/−^* mice (Figure 6A).

In a separate experiment, the progression of GM2 accumulation in double knockout mice was also assessed over five different time points: 8, 12, 16, 20 and 26 weeks of age (Figure 6D). There were no significant differences seen between any of the cohorts at any of the timepoints. It is possible that this experiment was not sufficiently powered to see differences in ganglioside levels throughout their lifetime.

**Figure 7:**
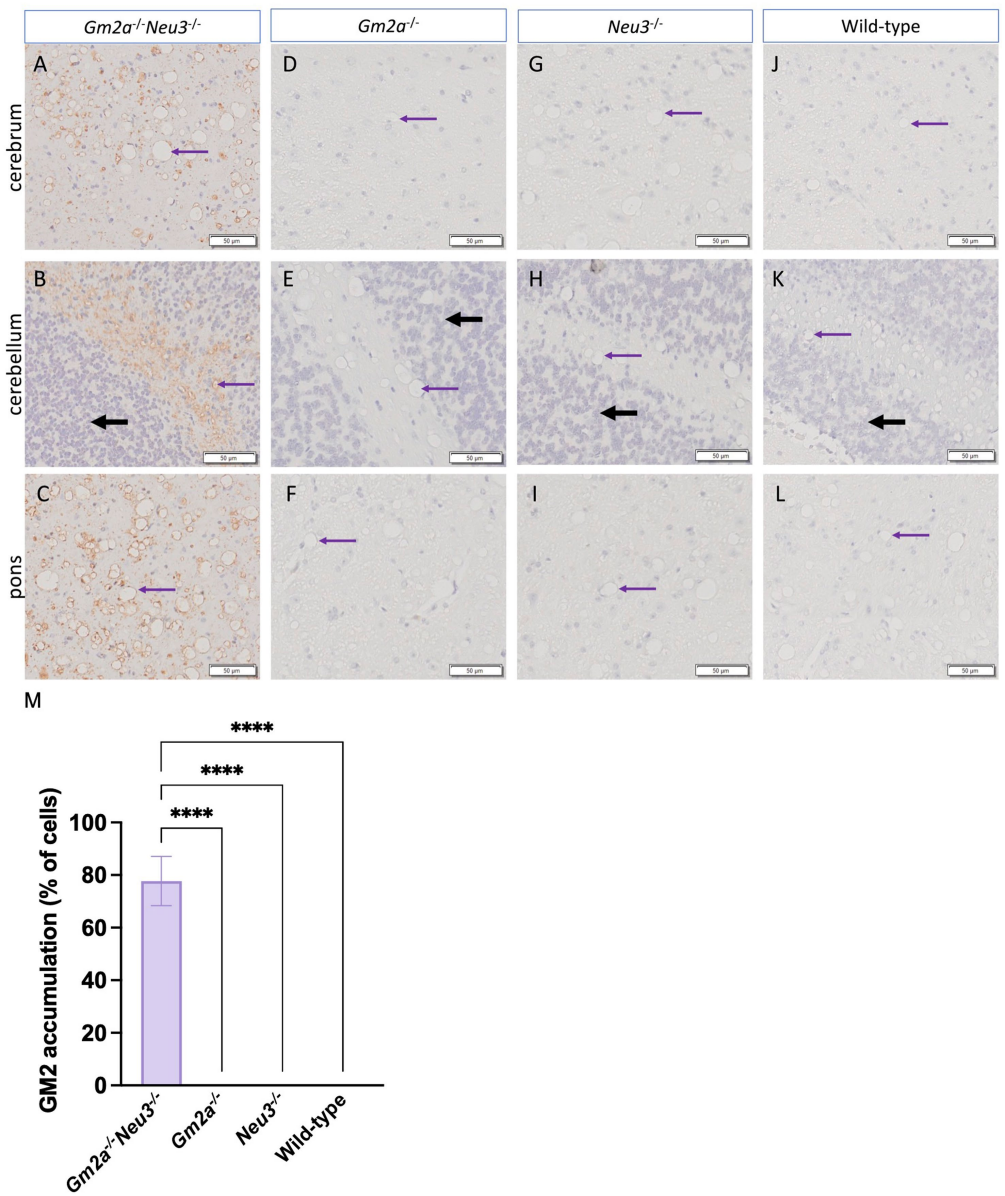
Histological analysis of ganglioside accumulation in the brain. Histological analysis to detect GM2 accumulation and vacuolization in the brains of *Gm2a*^−/−^*Neu3*^−/−^ mice. Brain sections were taken from the cerebrum, cerebellum, and pons of endpoint **(A–C)** *Gm2a*^−/−^*Neu3*^−/−^ (*n* = 2), **(D–F)** *Gm2a*^−/−^ (*n* = 3), **(G–I)** *Neu3*^−/−^ (*n* = 3), and **(J–L)** wild-type mice (*n* = 3) at 20× magnification. Sections were hematoxylin and eosin stained with an additional anti-GM2 antibody. The brown signal signifies GM2 in each of the sections, the black arrows indicate the granule layer, and the purple arrows demonstrate examples of vacuoles. GM2 accumulation was exclusively seen in *Gm2a*^−/−^*Neu3*^−/−^ mice at this time point. Additionally, vacuoles were observed in all cohorts but were most prominent in *Gm2a*^−/−^*Neu3*^−/−^ mice. **(M)** On average, *Gm2a*^−/−^*Neu3*^−/−^ mice (*n* = 2) had GM2 accumulation in 77.7% of their cells.

Histological analysis of murine brain sections supported our GM2 accumulation findings (Figure 7). The cortex (Figure 7A), cerebellum (Figure 7B) and pons (Figure 7C) in *Gm2a^−/−^Neu3^−/−^* mice display large accumulations of GM2 with no buildup visible in other cohorts. Accumulation in the cortex and pons is uniform across the entire section, whereas GM2 is almost exclusively built up in the white matter of the cerebellum (Figure 7). While GM2 is more abundant in the gray matter, it also has a major role in the development of myelin (Kraĉun et al., 1984; Sipione et al., 2020). Vacuolization is also visible in all histological brain sections of *Gm2a^−/−^Neu3^−/−^* mice but is most notable in the cerebrum and pons. These vacuoles appear to be larger and more abundant in all knockout cohorts compared to wild types.

## 4 Discussion

ABGM2 is the rarest of the GM2 gangliosidosis, with an estimated incidence rate of <1/1,000,000 and less than 30 reported cases, most of which are infantile onset (Sheth et al., 2016). The disease, with no current curative treatment, is believed to be underdiagnosed as there is not a consistent protein assay established for GM2A quantification in most clinical laboratories and the disease was less suspected/examined until recently when molecular testing with gene panels became available (Turro et al., 2020). To develop effective therapies, a representative animal model was essential. In this study, we knocked out *Neu3* in a *Gm2a*^−/−^ mouse, which is involved in the alternate catabolic pathway for GM2 ganglioside metabolism (Kolter and Sandhoff, 1998). The resultant double knockout mouse model (*Gm2a^−/−^Neu3^−/−^*) represents neuropathological and clinical abnormalities of a more severe form of ABGM2, compared to *Gm2a^−/−^* mice, and provides a valuable resource for future treatment development.

The *Gm2a^−/−^Neu3^−/−^* mice display a significant reduction in lifespan compared to *Gm2a^−/−^* mice (27 ± 1.5 weeks compared to 91.9 ± 9.8 weeks, respectively) (Figure 3). This shortened lifespan is likely attributable to the cytotoxic accumulation of GM2 (Figures 6, 7), which initiates a cascade of events within the CNS, including neuronal apoptosis and inflammatory responses (Demir et al., 2020). The onset of disease progression was observed at 12–16 weeks of age, evidenced by reduced coordination (Figure 4) and general locomotion (Figure 5). Therefore, knocking out *Neu3* in *Gm2a^−/−^* mice resulted in behavioral abnormalities, indicating a more severe pathogenesis than the previously characterized ABGM2 mouse model (*Gm2a^−/−^*) (Liu et al., 1997). Most mice exhibited body tremors, ataxia and total significant weight loss (Supplementary Figure 3) by their humane endpoint, similar to *Hexb^−/−^* mice (Phaneuf et al., 1996), albeit with a slightly longer life expectancy (27 weeks versus 16 weeks) and milder symptoms in early disease stages (Huang et al., 1997; Wada et al., 2000; Toro et al., 2021).

GM2 accumulation in *Gm2a^−/−^Neu3^−/−^* mice was comparable from 8 weeks of age to the animal’s humane endpoint (Figure 6D). In SD mice, GM2 accumulation is detected early in life; however, behavioral symptoms are not apparent until 12–16 weeks of age (Phaneuf et al., 1996; Hooper and Igdoura, 2016), suggesting that it is likely the downstream effects that trigger the progression of neurological decline in these animals. There is a sharp increase in microgliosis around 8–12 weeks of age, with subsequent astrogliosis around 12–14 weeks of age that coincides with increased caspase 9, a biomarker of apoptosis, along with other pro-inflammatory pathway (Hooper and Igdoura, 2016). Concurrent with astrocyte activation is a large decrease in motor function, suggesting that neuroinflammation is what drives disease pathology in GM2 gangliosidosis (Hooper and Igdoura, 2016). These timelines coincide with the decline in the behavioral parameters observed in *Gm2a^−/−^Neu3^−/−^* mice, which becomes most pronounced around 12 weeks of age and continues to deteriorate progressively (Figures 4, 5).

In the current investigation, a widespread accumulation of GM2 is observed in the white matter of murine brains, particularly in the cerebellar cortex (Figure 7). This accumulation is believed to also contribute to the phenotypic manifestation observed in behavioral testing, as disruption in the normal maturation of white matter can affect motor pathways, such as the pyramidal tract (Maegawa et al., 2009). Gangliosides play a major role in the development and stability of myelin (Schnaar, 2010; Vajn et al., 2013; Sipione et al., 2020), which is decreased in all GM2 gangliosidosis animal models (Maguire and Martin, 2021). Additionally, striking vacuolization is also visible in all histological brain sections of each cohort but is most notable in the cerebrum and pons of knockout mice. An abundance of vacuoles is characteristic of lysosomal storage disorders and has been previously observed (Pan et al., 2017).

The present study provides evidence that corroborates previous research regarding the involvement of NEU3 in ganglioside catabolism (Kolter and Sandhoff, 1998; Pan et al., 2017; Seyrantepe et al., 2018). The disruption of NEU3 protein activity resulted in an accelerated disease pathology, which is most likely attributed to the increased accumulation of GM2. However, if NEU3 were the sole sialidase involved in the metabolism of GM2, the phenotypic severity would match that of *Hexb^−/−^* mice (Phaneuf et al., 1996). When *Neu3* was knocked out in *Hexa^−/−^* mice, minor accumulation of GA2 was observed, indicating a possible involvement of another sialidase in this pathway (Seyrantepe et al., 2018). The alternate sialidase involved is likely NEU4, given that a loss-of-function mutation to this protein in *Hexa^−/−^* mice resulted in a more severe phenotype than that observed in single knockout Hexa*^−/−^* mice (Seyrantepe et al., 2010). Additionally, it is possible that in the absence of NEU3, both NEU4 and NEU1 may partially compensate for this sialidase loss, as all neuraminidase activity has been shown to be increased in mouse models of GM2 gangliosidosis (Seyrantepe et al., 2018).

We also found that knocking out *Gm2a* impacted expression of the β-subunits in the brain (Figures 2C,D). This is the first study to note these differences in expression as hexosaminidase isozyme activity is typically unaltered in ABGM2 models (Liu et al., 1997). An increase in expression of the β-subunit is observed in wild-type and *Neu3^−/−^* mice, but not *Gm2a^−/−^Neu3^−/−^* and *Gm2a^−/−^* mice. It’s possible that when the interaction between β-HEXA (αβ) and β-HEXB (ββ) and GM2A is altered, due to its absence, β-HEXA (αβ) and β-HEXB (ββ) are downregulated. β-HEXA enzyme activity was equal amongst all cohorts (Figure 2E), suggesting that the discrepancy in β-subunit expression is likely solely due to β-HEXB. However, there is a wide range of intra-cohort variability in the hexosaminidase assay, thus, further experimentation with a larger sample size is required to draw more concrete conclusions.

Our study presents compelling evidence regarding the involvement of NEU3 in GM2 metabolism and ABGM2 pathogenesis in mice. The newly developed *Gm2a^−/−^Neu3^−/−^* mouse model provides a more accurate representation of a severe form of the disease, both phenotypically and biochemically. These mice offer a unique opportunity to assess treatment effects on significant symptoms of the human disease, such as lifespan, coordination, gross locomotor activity and pathogenesis. As such, our findings hold great promise for future research on ABGM2 and potential treatments.

## Data availability statement

The original contributions presented in the study are included in the article/Supplementary material, further inquiries can be directed to the corresponding authors.

## Ethics statement

The animal study was approved by Queen’s University Animal Care Committee (2015-1603 and 2020-1959; 10/10/20). The study was conducted in accordance with the local legislation and institutional requirements.

## Author contributions

ND contributed to project administration, methodology, investigation, statistical analysis, figure development, and the writing and editing of the manuscript. CC, AR, BQ, and MM contributed to a subset of behavioral testing, blood collections, and euthanizations. PK ran the Western blots. AP donated the *Neu3*^−/−^ mice and offered guidance in genotyping. This study was conducted under the supervision of JW, who also designed the project and revised the final manuscript. All authors contributed to the article and approved the submitted version.
