# Supplementary material for: Characterization of a phenotypically severe animal model for human AB-Variant GM2 gangliosidosis
Source: Front Mol Neurosci. 2023 Nov 30;16:1242814. doi: 10.3389/fnmol.2023.1242814 (PMC10720325; doi:10.3389/fnmol.2023.1242814)
Supplement: Supplementary file 1 [file Data_Sheet_1.docx]

Supplementary Material

Characterization of a Phenotypically Representative Animal Model for Human Juvenile-Onset AB-Variant GM2 Gangliosidosis

Natalie M. Deschenes^*^, Camilyn Cheng, Prem Khanal, Brianna M. Quinville, Alex E. Ryckman, Melissa Mitchell, Alexey V. Pshezjetsky, Jagdeep S. Walia

*** Correspondence:** Natalie M. Deschenes; 12nmd4@queensu.ca

# Supplementary Data

## Supplementary Figures


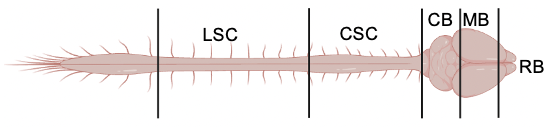


Supplementary Figure 1. Sectioning of the brain and spinal cord. Schematic of the brain sectioning at euthanization. LSC: lumbar section of the spinal cord. CSC: cervical section of the spinal cord. CB: caudal section of the brain. MB: mid-section of the brain. RB: rostral section of the brain.

Supplementary Figure 2. Genotyping Gels for *Gm2a* and *Neu3.* (A) Gel electrophoresis of DNA amplified by PCR using *Neu3* primers against a wild-type and knockout mouse sample. Lane 1 is a 1kb ladder. Lane 2-3 are samples ran with forward and reverse primers for wild type *Neu3.* Lane 4-5 are samples ran with forward and neomycin cassette primers depicting a knocked out *Neu3*. The wild-type band is 2110bp and the knockout band is 1689 bp. (B) Gel electrophoresis of DNA amplified by PCR using *Gm2a* primers against a wild type and knockout mouse sample. Lane 1 is a 100bp ladder. Lane 2-3 uses a combination of forward, reverse and neomycin cassette primers for *Gm2a*.

Supplementary Figure 3. Symptom onset and disease manifestation. (A) Symptom onset and death of *Gm2a^-/-^Neu3^-/-^* mice had a 78.61 correlation (p<0.0001). Thus, the earlier symptoms were noted in *Gm2a^-/-^Neu3^-/-^* mice, the earlier they reached their humane endpoint. On average, *Gm2a^-/-^Neu3^-/-^* mice reached their endpoint within 1.5-3 weeks of their first presenting symptom. (B) Disease manifestation observed in *Gm2a^-/-^Neu3^-/-^* mice. The percent of the population (n=29) that presented with the various phenotypes observed at end-of-life (24-30 weeks of age) are shown. Overall, *Gm2a^-/-^Neu3^-/-^* mice were often shaky and ataxic by their humane endpoint. Additionally, many of the mice also experienced extreme weight loss, which was defined as losing over 15% from their peak weight.
